# Shear behavior of bedding fault material on the basal layer of DGB landslide

**DOI:** 10.1038/s41598-023-27488-5

**Published:** 2023-01-05

**Authors:** Yufei Liang, Shenghua Cui, Xiangjun Pei, Ling Zhu, Hui Wang, Qingwen Yang

**Affiliations:** State Key Laboratory of Geohazard Prevention and Geoenvironment Protection, Chengdu University of Technology, Chengdu, 610059 China

**Keywords:** Hydrology, Natural hazards

## Abstract

Daguangbao (DGB) Landslide (12 × 10^8^ m^3^) is the largest landslide triggered by the 2008 Ms8.0 Wenchuan Earthquake, in which basal shear failure develops on an interlayer fault at 400 m deep under the ground. After the landslide, a 1.8 km long (in the sliding direction) oblique shear face is exposed. Different kinds of materials in the interlayer fault of DGB landslide are taken for direct shear test, medium scale shear test, in-situ shear test and ring shear test. The test results show that fault material cohesion ranges from 20 to 320 kPa and internal friction angle from 15° to 41°. Shearing strength of interlayer fault materials is related to fragmentation degree of structure. The lower fragmentation degree the more obvious strain softening characteristics of materials, the higher fragmentation degree the poorer shearing resistance of materials. Compared with argillaceous materials in the same fault, the mylonitic materials are of higher shear strength and internal friction angle. Both mylonitic materials and breccia materials are strong in liquefying. In saturated undrained cases, shear strength of fault materials could drop to 9.7°, with S3 down to 0. In saturated undrained dynamic shear conditions, fault internal friction angle could be reduced to 23.1° and 4.2°. It is concluded that low friction feature of fault materials caused by the influence of groundwater is the main reason for destabilization of DGB landslide.

## Introduction

DGB landslide is the largest landslide triggered by the 2008 Ms8.0 Wenchuan Earthquake, of which the area is 7.1 km^2^, maximum accumulative thickness about 600 m, length 4.6 km, width 3.7 km and estimated volume 12 × 10^8^ m^3^^1^. To the south of its sliding source area, there is a 1.8 km long (in sliding direction) oblique shear face with the area of 0.3 km^2^. Huang has carried out research on the DGB landslide first and proposes that the landslide is the coupling result of earthquake, geology and landform^2^. By FLAC^3D^ process simulation, Yin et al. discovers that horizontal seismic acceleration could promote the release of steep vertical plane of the Landslide^3^. Other researchers including Zhang et al.^4^, Song et al.^5^, and Wu et al.^6^ find that vertical shaking makes great contributions to DGB landslide according to the discontinuous deformation analysis (DDA) results. Cui et al. have conducted 12 shaking table tests on the block model with saturated soft intercalated layer^7,8^. They deduct from the test results that earthquake vibration brings about transient load to the weak layer of the slope, so the weak intercalated layer suffers not only greater compression force but also greater tensile force (more than 30 times that on upper and lower hard layers). The increasing tensile stress and shear stress results in the generation of dynamic cracks in weak layers according to Pei et al.^9^. This opinion could be verified by the large number of cracks (hundreds of cracks/m^2^) newly developed in drilling holes on ditches, tunnels and DGB landslide dynamic face.

Researchers mainly focus efforts on shear strength of rock on sheared face^10–13^. Liu et al. reveal that friction weakening of sheared face is an important reason for the characteristic of remote movement of DGB landslide by the modified DAA^14^. By tunneling (2 m long) at the foot of south scarp of landslide, Cui et al.^7^ and Pei et al.^9^ suggest that a 3 m-thick bedding fault mainly composed of breccia is the main geological structure to landslide formation. The fault is 400 m deep under the ground. Shear failure of DGB landslide happens here in the fault. Zhang et al. has obtained the calibrated friction coefficient^4^ (corresponding to the friction angle of 10°), which is 0.18, by DDA based numerical simulation and some parameters restrained by the final geometrical shape of landslide deposits. These friction coefficients could be used to explain the uncontrolled instability of the landslide in the sliding direction. It is observed from rotary shear test that friction coefficient in steady state changes between 0.05 and 0.16, which could be used to explain the long run-out of DGB landslide^15,16^.

In the newly dug tunnel (24.5 m long), it is found that there is groundwater flowing in the bedding fault of DGB landslide. Breccia in the bedding fault has improved permeability of fracture zone (namely the zone parallel with structure). Groundwater flow rate in tunnel is about 13–18 L/h/m^2^ according to the founds. Although fault-rated hydrogeology is an important engineering geology element to landslide formation, neither groundwater conditions nor saturation conditions of bedding fault are taken into consideration in the previous test and numerical simulation.

Therefore, the possible functions of saturated fault breccia to the formation and movement of DGB landslide are still unclear. To illustrate the friction characteristics of DGB landslide Zone, in-situ shear test, indoor direct shear test and indoor ring shear test are conducted under dry and wet conditions. In this paper, efforts are mainly focused on studying the shear characteristics of rock in bedding fault and learning about the impacts of fragment degree of bedding fault materials and groundwater to shear behaviors.

## DGB Landslide

**Figure 1:**
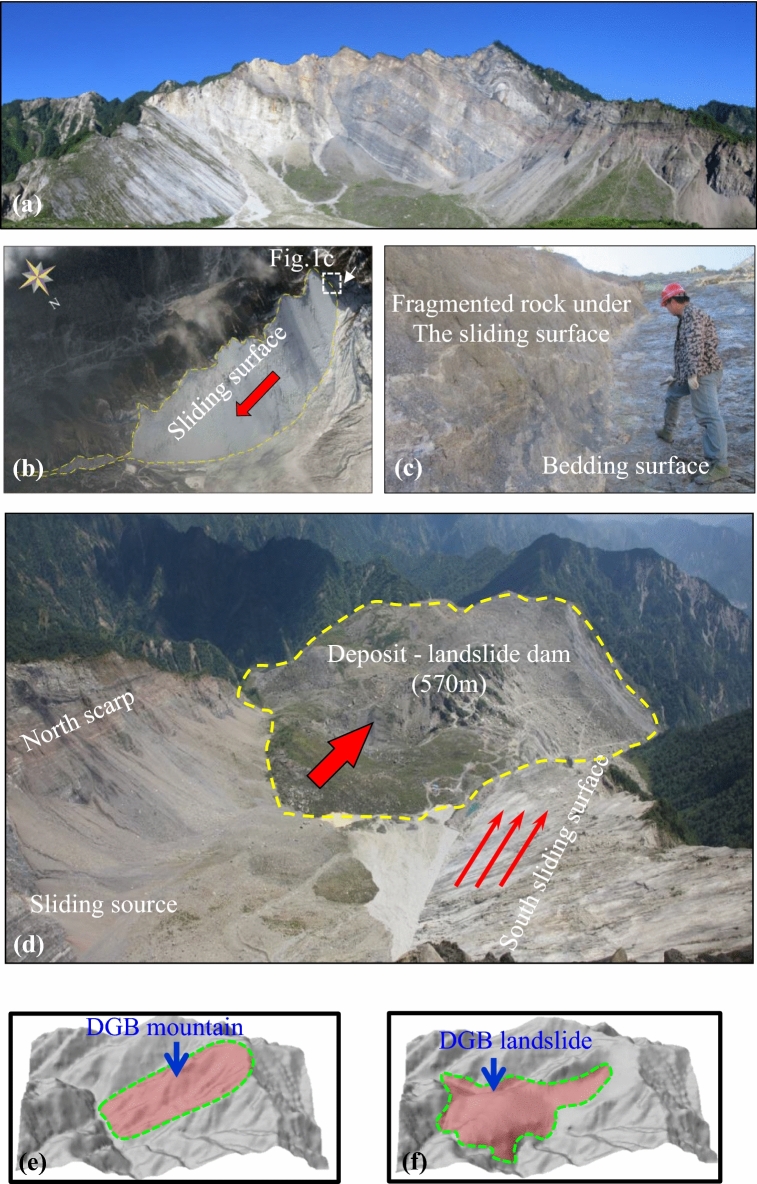
Geological setting of the sliding zone of DGB landslide (**a**) Overall view of DGB landslide; (**b**) South sliding surface of DGB landslide; (**c**) section showing the fault rock overlaying the bedding plane; (**d**) Top view of DGB landslide deposit area, the thick red arrow shows the direction of landslide movement, the thin red arrow shows the sliding direction of the south sliding surface. (**e**) Topography of the DGB mountain before landslide (**f**) Topography of DGB landslide.

Located in Gaochuan Township, Anxian County, Mianyang City, China, DGB landslide is 4.5 km from the causative fault to the 2008 Wenchuan Earthquake, Yingxiu-Beichuan Fault. The area where DGB landslide is located belongs to Dashuizha Nappe, in which NW–SE reverse fault system and NE anticlinal are developed^17^. Within the area of landslide, it is mainly covered by carbonate rock outcrops. Rear edge and both sides of DGB landslide are featured with a large area of the exposed stratigraphic section, on which the stratigraphic contact relationship and layer thickness are very clear (Fig. 1).

There are three groups of dominant joint sets in the research area. Group I is moderately dipping rock layer with the attitude of N88°W/NE∠32°. Group II is NE strike, SE inclined crack (J1) with the attitude of N58°E/SE∠72°. And Group III is NW strike, NE inclined crack (J2) with the attitude of N10°W/NE∠84°. These three joint sets are the geological basis for boundary conditions of DGB landslide. Under the shearing effects of bedding plane and the developed two joints J1 and J2, the slope is developed with huge potential unstable wedge sliding mass and tends to move along stratum strike.

**Figure 2:**
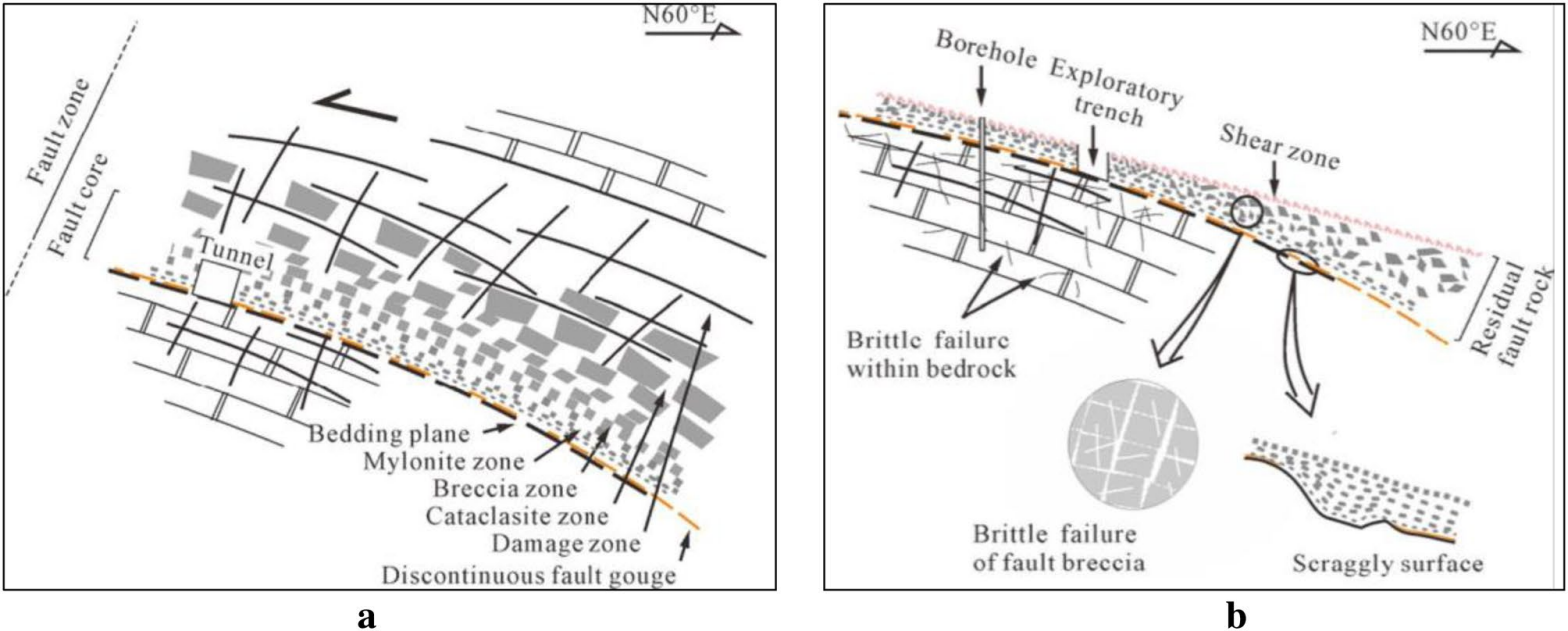
Geological model of the sliding zone of DGB landslide (**a**) Before DGB landslide; (**b**) After DGB landslide (Modified from Cui et al.^18^).

Fault rock with the average thickness of 3 m is left over the shear face of DGB landslide. Cui et al.^18^ divides the broken area by rock compositions from bottom to top, which include the 0.2–5.0 cm slightly wet soft plastic argillaceous zone, 3.0–45.0 cm mylonitic zone containing a small amount of dolomite and argillaceous lens, the grayish-white calcite cemented breccia zone and fragmented rock zone with lots of visible dolomite lens, as well as the fragmented rock zone affected by faults (Fig. 2).

## Methods

### Sample

**Figure 3:**
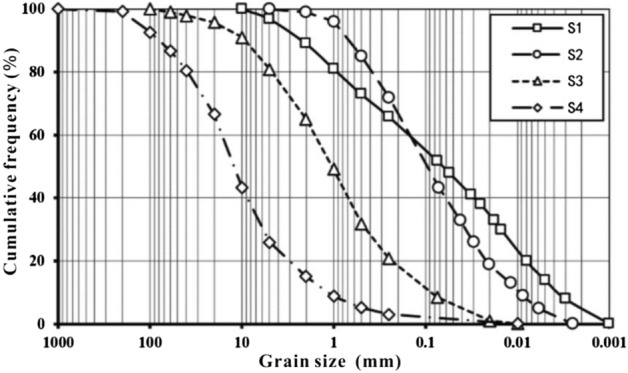
Grain-size distribution curves of the samples.

Making use of the indoor direct shear apparatus, on-site large scale shear apparatus and the ring shear apparatus of the Disaster Prevention Research Institute (DPRI), shearing mechanical behaviors of sliding zone soil are studied in this paper. Take the argillaceous materials (S1), mylonitic materials (S2), fine breccia materials (S3) and coarse breccia materials (S4) in bedding fault as the test materials. S1 contains 56% of dolomite and 42% of clay minerals. While S2, S3 and S4 mainly contain 90–95% dolomite and a small amount of quartz and kaolinite. Contents of clay particles (< 0.075 mm) in S1 and S2 are respectively 53% and 42%, while the clay particle size of S2 is closer to the upper limit. S3 particle size is between 0.25 and 5 mm and S4 particle size is larger than 5 mm (Fig. 3). Based on the nonuniform coefficient data that S1 is 40.2 at maximum, S2, S3 and S4 are 15.6, 17.6 and 14 respectively, the four kinds of materials could be rated with the grading effects of S1 > S3 > S2 > S4, which means that the higher the nonuniform coefficient, the better grading effect. For natural water content and dry density, S1 is 12% and 2.13 g/cm^3^, and S2 is 8% and 2.32 g/cm^3^. The liquid and plastic limits of S1 are 24% and 11.8%, and those of S2 are 12% and 7%. The natural densities of S3 and S4 are 2.51 g/cm^3^ and 2.53 g/cm^3^.

### Direct shear test

Direct shear test covering simple direct shear test and medium scale shear test takes S1 and S2 of smaller particle sizes as the test objects. During simple direct shear test, the strain-controlled direct shear apparatus is applied for fast shear test. At first, prepare five samples with different natural water contents for each material according to their liquid and plastic limits as follow: 8%, 12% (natural moisture content), 16%, 20% and 24% for S1 and 4%, 6%, 8% (natural moisture content), 10% and 12% for S2. Then, apply the same normal stresses to both materials, which are 100, 200, 300, 400 and 500 kPa.

The shear box for medium scale shear test is in the size of 14 cm × 15 cm × 16 cm (L × W × H). Test objects are S3 and S4 of larger particle sizes and S2 used for comparison. Control sample density according to natural material density, keep normal stress unchanged during test, increase shear stress gradually and record corresponding shear strain. When shear stress exceeds the peak strength, sample is broken and shear strain increases rapidly. Stop test when shear displacement exceeds 1 cm (namely strain reaches or exceeds 6%). The range of normal stress is set between 0.3 and 1.5 MPa. Divide the normal stress range into 6 levels for tests.

### In-situ shear test

**Figure 4:**
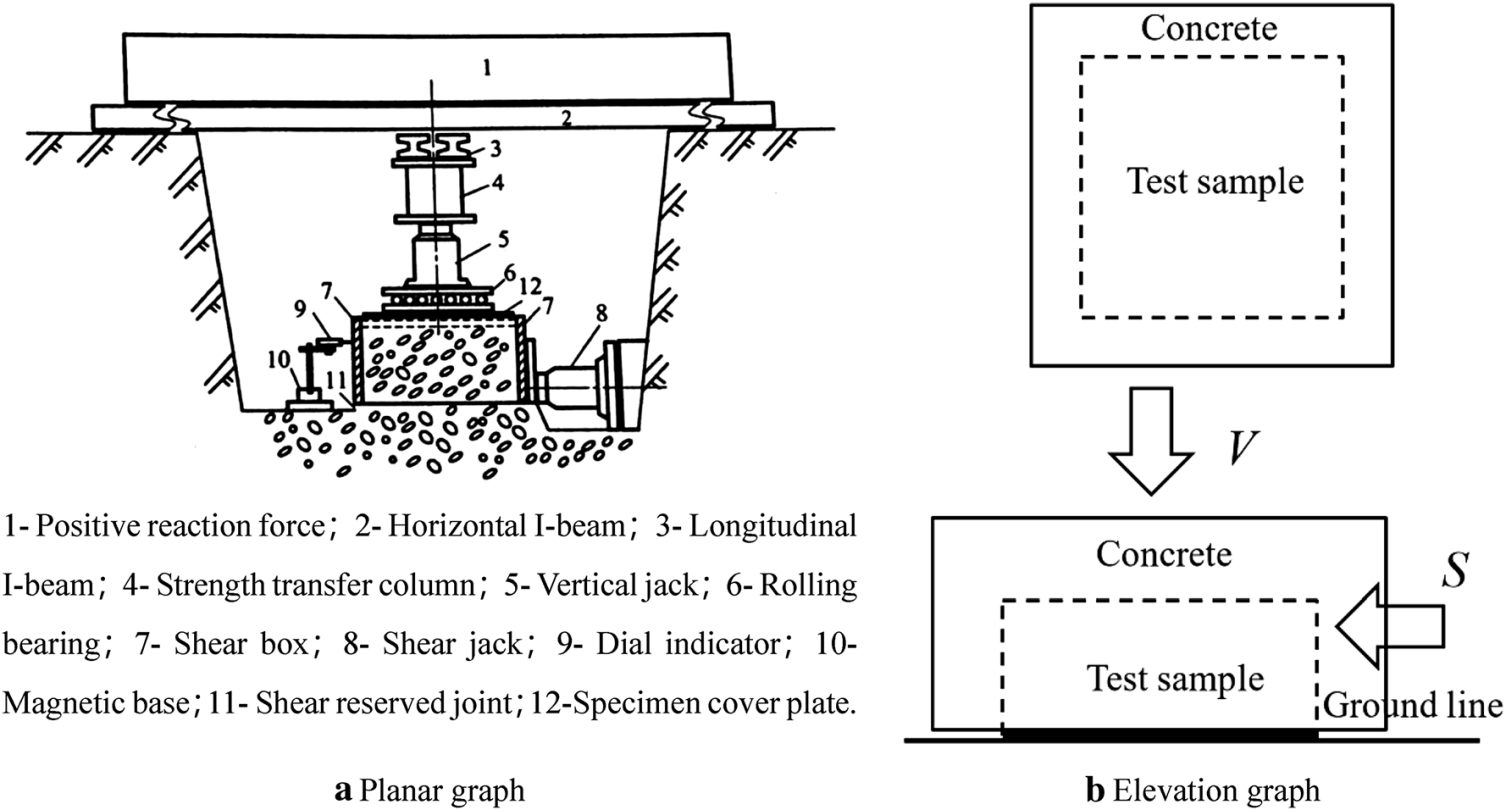
Device of in-situ shear test.

To do in-situ shear test on mylonitic zone of fault, cut rock mass into 6 parts in the same size of 0.5 m × 0.5 m × 0.2 m (L × W × H) (1#, 2#, 3#, 4#, 5#, 6#), and pour 0.1 m thick concrete around samples as protection shield (Fig. 4).

**Figure 5:**
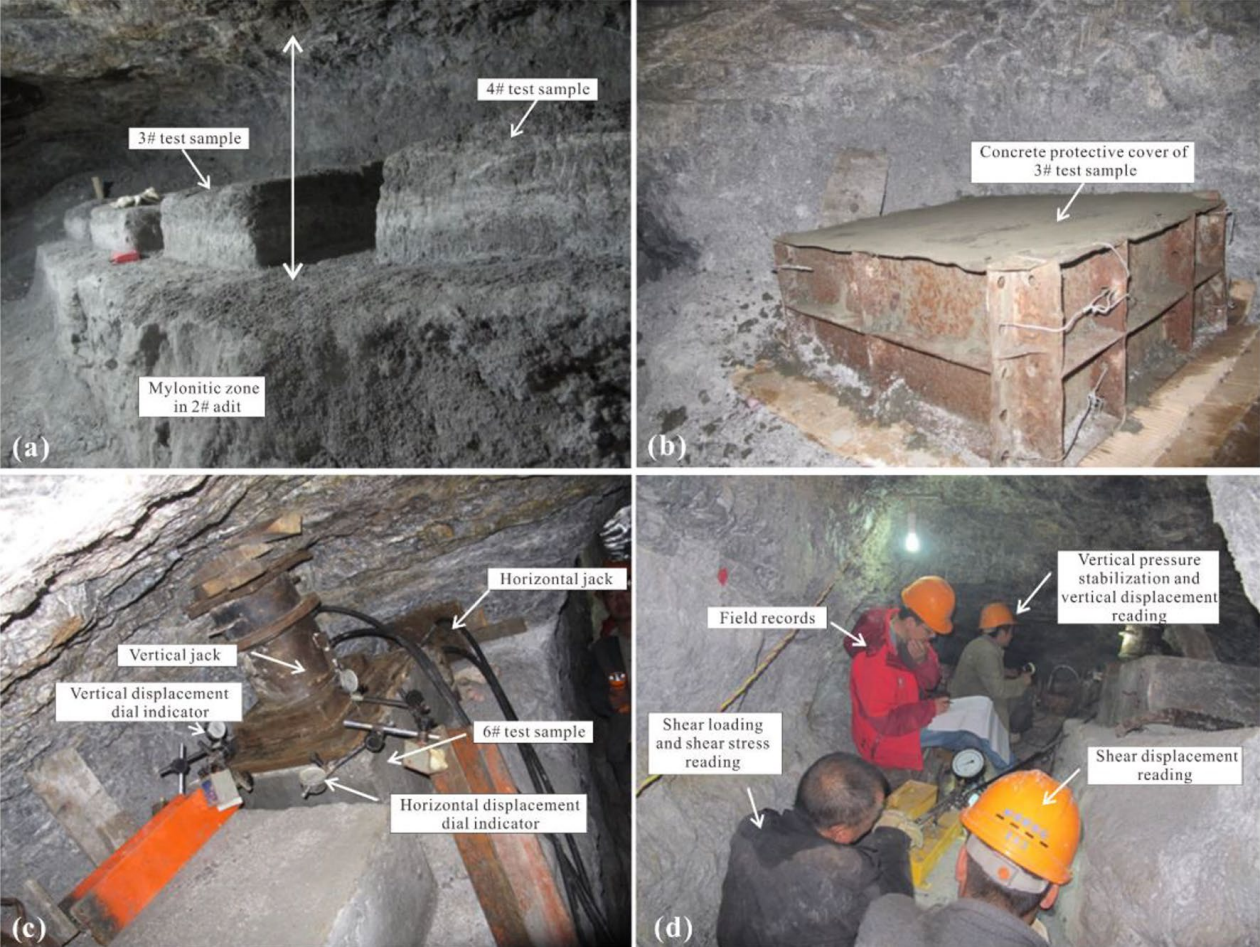
Process of in-situ shear test (**a**) Field pictures of 3# and 4# test samples, and mylonitic zone in adit; (**b**) Concrete protective cover of 3# test sample; (**c**) Device of in-situ shear test and 6# test sample; (**d**) Field record of in-situ shear test.

Apply load on samples through vertical and horizontal jacks. Remain the vertical loads (*V*) on the same sample unchanged at 50 kN, 100 kN, 125 kN, 175 kN, 220 kN and 400 kN respectively. The stresses on the section of corresponding samples are 0.2 MPa, 0.4 MPa, 0.55 MPa, 0.69 MPa, 0.88 MPa and 1.6 MPa. As cross section and side face of one sample are perpendicular to each other, shear force would not affect positive force^19^, it is applicable to impose shear force (*S*) on side face of sample by horizontal jack. In the beginning of shear test, increase the shear force to 10 kN, then hold for 5 min, and go on increase it until the sample is broken (Fig. 5).

Shear test contains two parts, cut off test and the shear test on the sheared plane. Turn over the sample to record the characteristics of the upside and downside of the sheared plane and measure its actual area after test. Calculate the normal stress (σ) and shear stress (τ) on the sheared plane based on the forward and horizontal loads and actual shearing area. And draw the relationship curve of σ and τ. In the end, confirm shear strength of rock mass (joint face) by Coulomb Equation.

### Ring shear test

**Figure 6:**
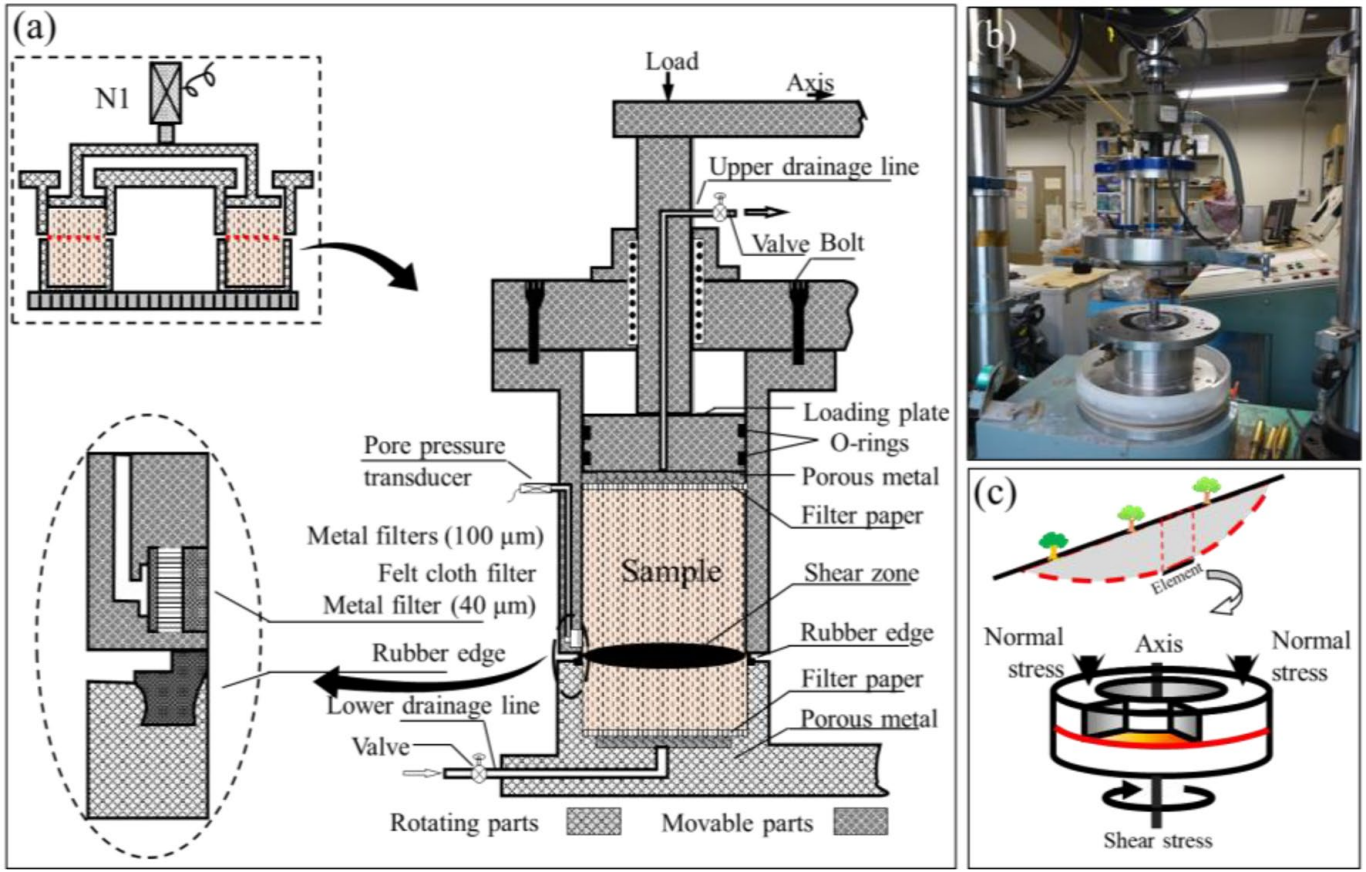
Configuration of DPRI 5 ring shear apparatus (**a**) The design of undrained ring shear box a The conception of ring shear test; (**b**) Ring shear test equipment; (**c**) The conception of ring shear test (Modified from Cui et al.^20^).

DPRI ring shear apparatus (Fig. 6) could be used to simulate the destroying process of soil units inside geological body and shearing behavior during long-distance movement. Undrained shear box is an important part for the apparatus. The shear box is divided into upper and lower halves in the middle. The internal annular space is sample room. The rubber materials of high wear resistance for inner and outer rings of lower half could ensure the enclosure of sample room under positive pressure. The upper half is designed to give normal stress to sample. Driven by lower half, shear box rotates on rubber mat and cut the sample. The drain valve in lower part could change the shearing condition between drained state and undrained state freely. During test, it is applicable to test the pore pressure in shear zone, shear displacement, shear stress and normal stress. For the dimensions of the shear box, its inner diameter is 120 mm, outer diameter 180 mm and height 115 mm.

To remodeling soil in sliding zone, break S1, S2 and S3 of sliding zone into pieces with wood hammer before drying them in 100 °C oven, then screen out the 4.75 mm particle samples. Items greater than 4.75 mm in S1, S2 and S3 respectively take up 1.2%, 5.35% and 15.6% of the total amount of sample. It is believed by Kuenza et al.^21^ that, shear behavior is mainly subject to the effects of materials smaller than 4.75 mm when the content of 40 gravel (> 4.75 mm) lower than 40%. So removing gravels changes little on the test results.

After sample preparation, put samples into shear box by dry settling^22^. To make the sample evenly distributed, use hopper for setting out. Add normal stress to samples from the top by loading plate and inject CO_2_ and water into the box through valve at the bottom to saturate the samples. Check the saturation regime of sample by *B*_D_ test^23^ as follow. Open up the drainage valve first on the top, give normal stress to remove pore water pressure. Close the valve after pore pressure drops to 0. Increase the normal stress in undrained conditions and measure the increment of pore water pressure. Calculate *B*_D_ by the following equation:

1 $$ B_{{\text{D}}} = \Delta u/\Delta \sigma $$

Wherein *Δu* is the increment of pore pressure, *Δσ* is the increment of total normal stress. If *B*_D_ is greater than 0.95, it meets the saturation requirements. Then give consolidation stress to consolidate the samples. If vertical displacement remains unchanged, the consolidation must have been finished.

After consolidation, give 200 kPa normal stress to S1, S2 and S3 and keep the normal stress the same. Then add shear stress at a constant rate of 0.5 kN/s. When shear stress exceeds shear strength, shear failure occurs and shear displacement increases rapidly. Stop test when shear stress stops increasing, as sample has reached the residual shear strength.

Ring shear test includes saturated drainage shear test and undrained static shear test, the test process of which is almost the same. While saturated drainage shear test needs to open the drainage valve during shearing to expel excess pore water (if any). According to the requirements, S1 and S3 are put to the undrained static shear test, and S2 is put to both the saturated drainage shear test and the undrained static shear test.

## Results

### Direct shear test data

#### Simple direct shear test

As moisture content increases, S1 increases in tangent modulus and decreases in shear strength, while S2 decreases in both tangent modulus and shear strength. The difference in strength may reduce deformation effect when pore pressure generated during undrained fast shear condition cannot be timely released because that S1 is greater than S2 in contents. Curve of S1 is strain hardening type curve, while curve of S2 turns into strain softening type after moisture content of S2 reaches a certain level.

Under the normal stress, the stress–strain curves of S1 and S2 meet the straight line Mohr Coulomb Criterion (Fig. 8a), $\tau = \sigma \tan \varphi + c$. For fault argillaceous materials, the cohesion and inner friction angle are 0.0214 MPa and 17°. For mylonitic materials, the cohesion and inner friction angle are 0.0249 MPa and 32°. Take the parameters into following equation:

2 $$ \left\{ \begin{gathered} \tau_{s1} = 0.3\sigma + {0}.{0}214 \hfill \\ \tau_{s2} = 0.62\sigma + 0.{0}249 \hfill \\ \end{gathered} \right. $$

Wherein $\tau_{s1}$ and $\tau_{s2}$ are shear strengths (MPa) of S1 and S2 respectively.

#### Medium scale shear test

**Figure 7:**
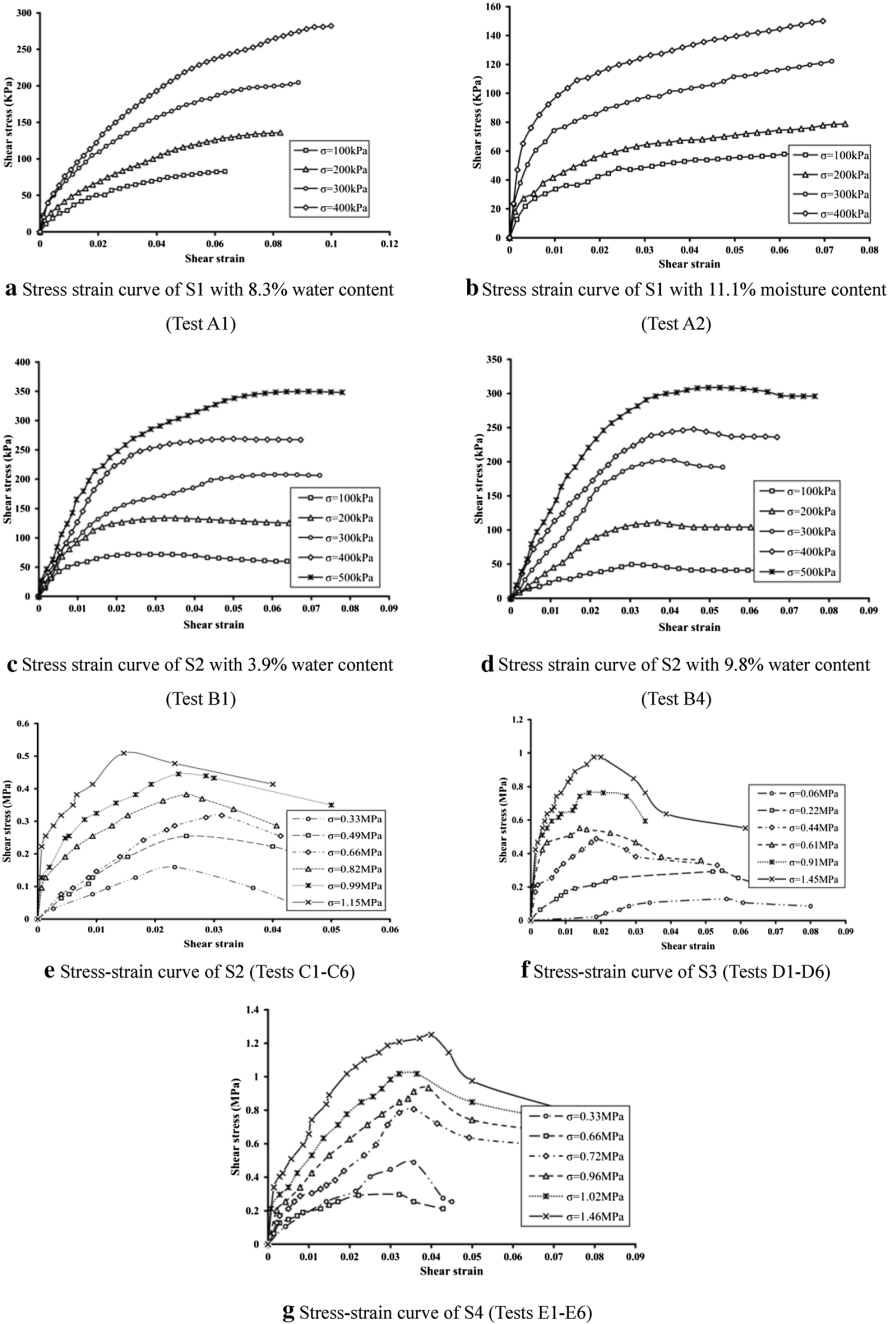
Stress–strain curves of simple direct shear tests (**a**–**d**) and indoor medium shear tests (**e**–**g**).

S2 shows a certain degree of strain softening effect (Fig. 7). S3 has no obvious peak under low normal stress and turns into strain softening gradually as normal stress increases. S4 apparently shows the strain softening characteristics. It might because that S4 particles are relatively bigger, the mosaic structure between particles could bear greater shear stress. Once the shear strength is exceeded or permissible gradient is reached, sample would be broken out of blue and stress would decrease. It is worth noting that S3 is smaller than S4 in pre-peak strain, which means that S3 is of greater shear modulus. According to particle gradation results, it could be known that the non-uniformity coefficient of S3 is greater than S4, which not only realizes a closer particle contact of S3, but also enlarges its shear modulus.

**Figure 8:**
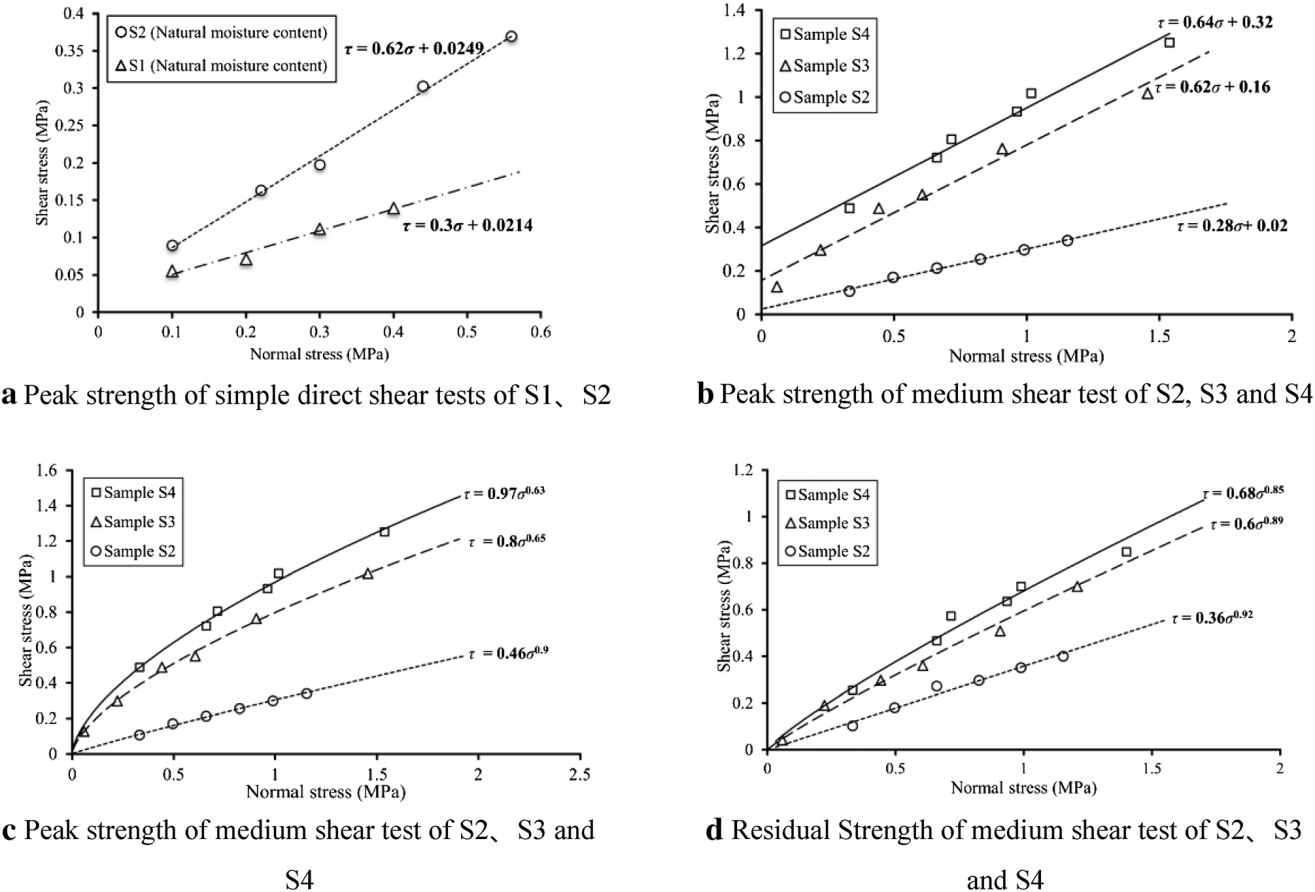
Strength envelope of indoor direct shear test.

By doing straight line Mohr Coulomb fitting for S2, S3 and S4 (Fig. 8b),$\tau_{s2} = 0.28\sigma + {0}.02$, $\tau_{s3} = 0.62\sigma + {0}.16$, $\tau_{s4} = 0.64\sigma + {0}.32$, it is observed that the inner friction angle and cohesion of S2 in medium scale shear test are smaller than simple direct shear test results. Shear strength of fault materials is of non-linear characteristics^24^. And S3 and S4 are more appropriate for the power function relation $\tau = a\sigma^{b}$ under test stress level. Then do the fitting calculations for peak and residual strength separately by the equations as follow (Fig. 8c,d):

3 $$ \left\{ \begin{gathered} \tau_{s3} = 0.8\sigma^{0.65} ,\tau_{s3}^{^{\prime}} = 0.6\sigma^{0.89} \hfill \\ \tau_{s4} = 0.97\sigma^{0.63} ,\tau_{s4}^{^{\prime}} = 0.68\sigma^{0.85} \hfill \\ \end{gathered} \right. $$

Wherein, $\tau_{s3}$ and $\tau_{s3}^{^{\prime}}$ are peak and residual shear strength (MPa) of S3 and $\tau_{s4}$ and $\tau_{s4}^{^{\prime}}$ are peak and residual shear strength (MPa) of S4.

### In-situ shear test data

#### Sheared section characteristic

**Figure 9:**
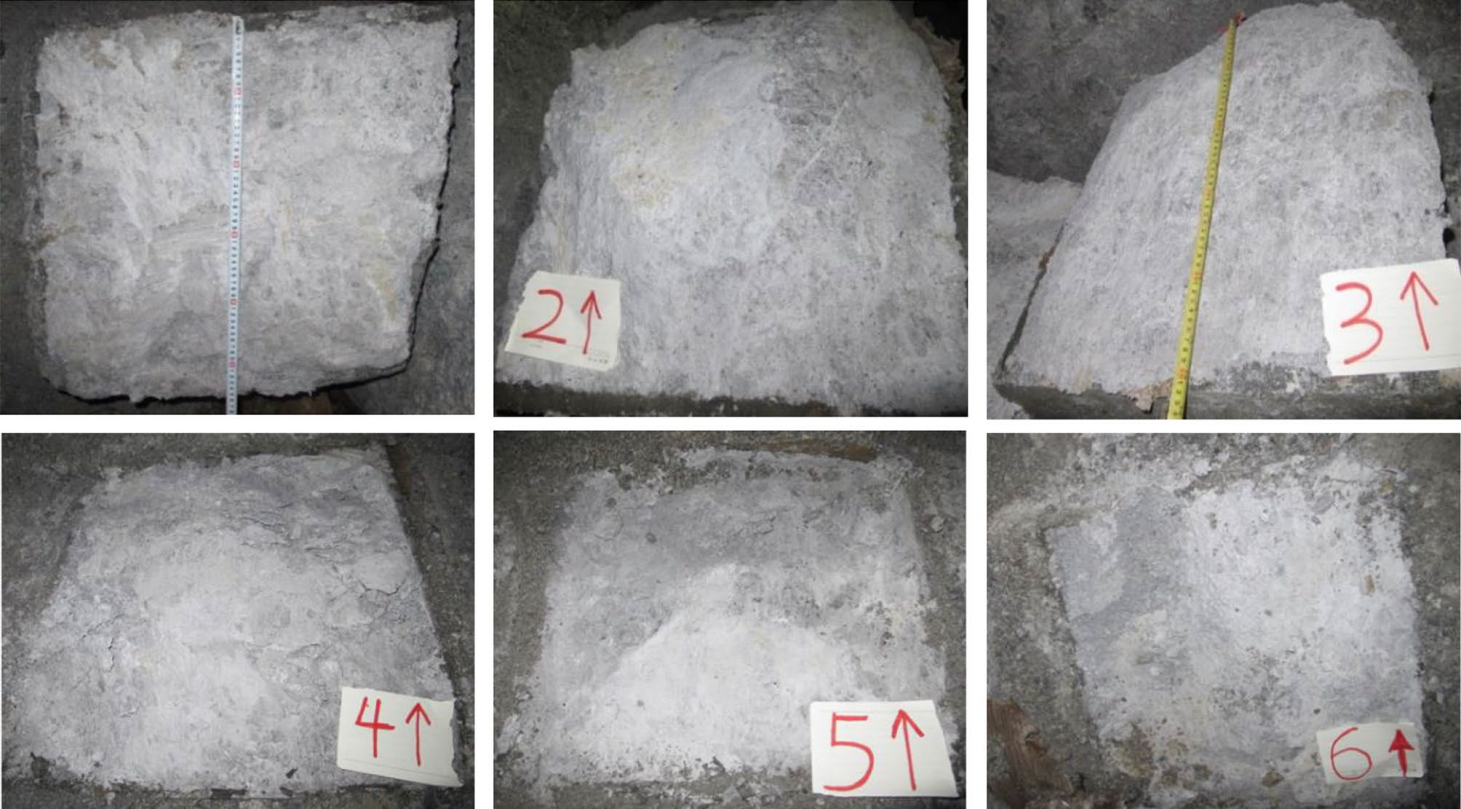
Pictures of surface after the first shear test.

Under low normal stress (Samples 1#, 2# and 3#), sheared face goes up and down slightly and is little correlated with normal stress. It basically stretches along the preset plane with the maximum fluctuation range of 1.5 cm (Sample 2#). With the increasing of normal stress (Samples 4#, 5# and 6#), both the range and frequency of fluctuation rise. Under the normal stress of 1.6 MPa, the fluctuation range reaches the peak (Sample 6#) at 5 cm (Fig. 9).

**Figure 10:**
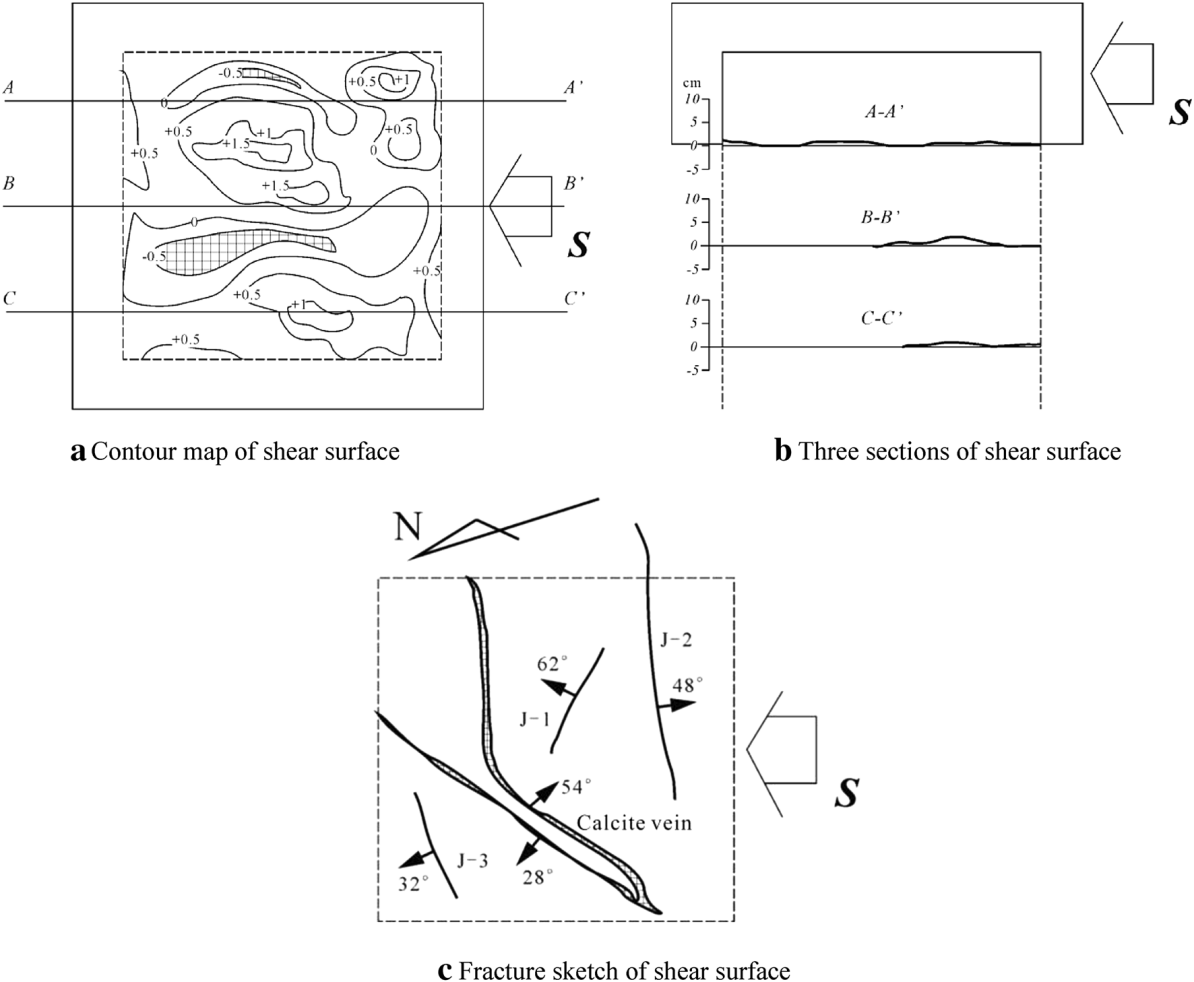
Characteristics of shear surface.

According to the crack sketch of sheared face (Fig. 10), it is obtained that sample is surrounded by three cracks (J-1, J-2 and J-3) which incline to NE, SSW and NNW respectively. Wherein, J-1 show the steepest dip angle while J-3 the lowest. In the middle, there is developed with a calcite vein across the sheared face.

#### Shearing deformation and strength

**Figure 11:**
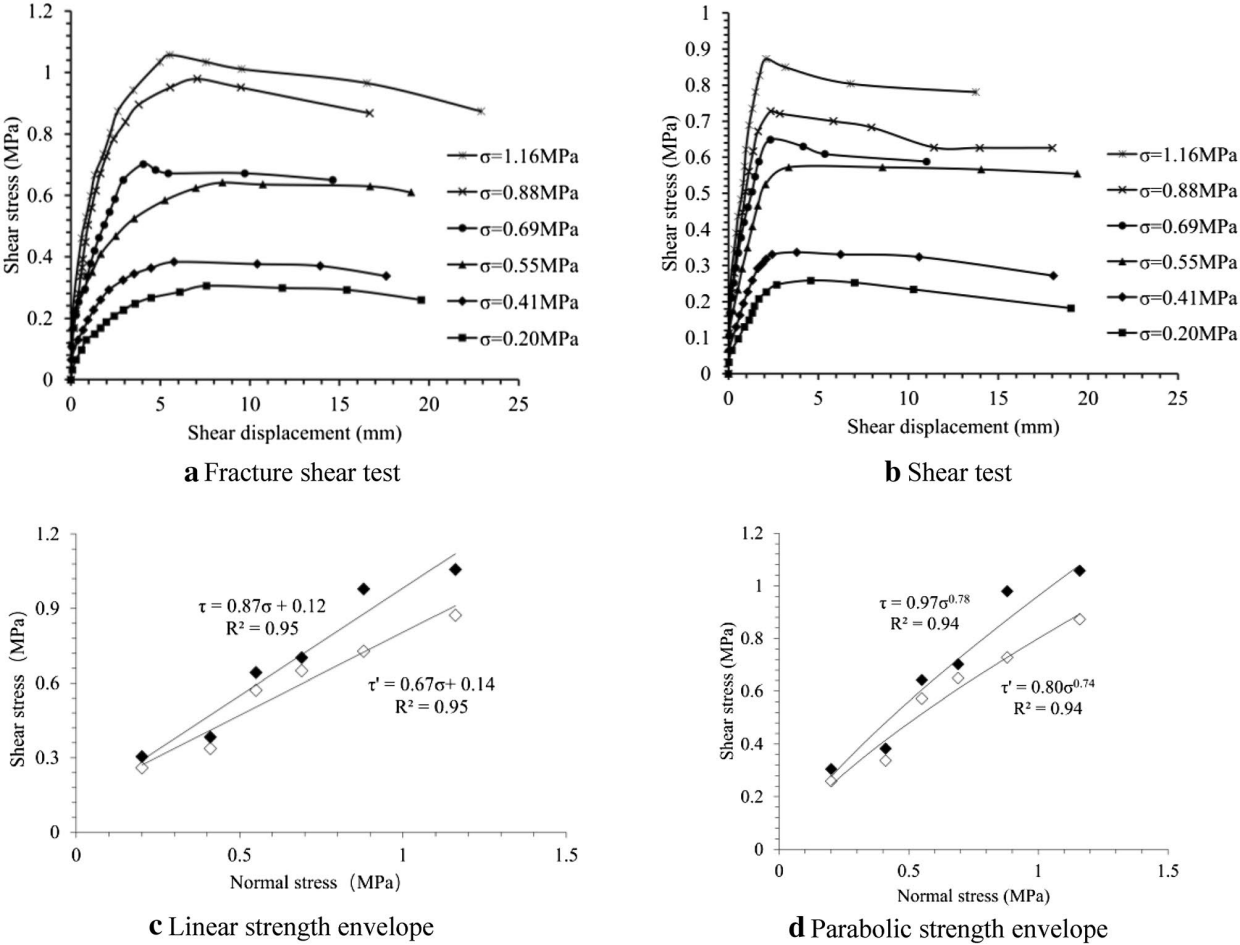
Shear normal stress and strength.

During the first shearing, the stress–strain curve of rock mass in sliding zone has no obvious peak under low stress status. When the normal stress reaches 0.69 MPa, the peak occurs (Fig. 11a). As normal stress getting larger, peak value increases and post-peak stress drop becomes greater. During the second time, peak only occurs when the normal stress reaches 0.88 MPa (Fig. 11b) and post-peak stress drop is much more obvious than the first time. Post-peak shear displacement increases, but shear stresses of all samples are almost the same, which can be regarded as that residual value has been reached. As normal stress magnifying, peak strength is enhanced. As the second shear is based on the first one, the peak strength of sample at second time is lower than that in the first time. Do fitting calculation on the two shear peak strengths by straight line Mohr Coulomb Criterion (Fig. 11c), the results are shown as follow:

4 $$ \left\{ \begin{gathered} \tau = 0.87\sigma + 0.12 \hfill \\ \tau ^{\prime} = 0.67\sigma + 0.14 \hfill \\ \end{gathered} \right. $$

Wherein: $\tau$ and $\tau ^{\prime}$ are the peak strengths of clipping and shearing, of which corresponding inner friction angle and cohesion are 41° and 0.12 MPa for the first shear, 33.8° and 0.14 MPa for the second respectively. Then do parabolic type fitting calculation on the two shear peak strengths by Mohr Coulomb Criterion (Fig. 11d), and the results are shown as follow:

5 $$ \left\{ \begin{gathered} \tau = 0.97\sigma^{0.78} \hfill \\ \tau ^{\prime} = 0.8\sigma^{0.74} \hfill \\ \end{gathered} \right. $$

#### Shear dilatancy

**Figure 12:**
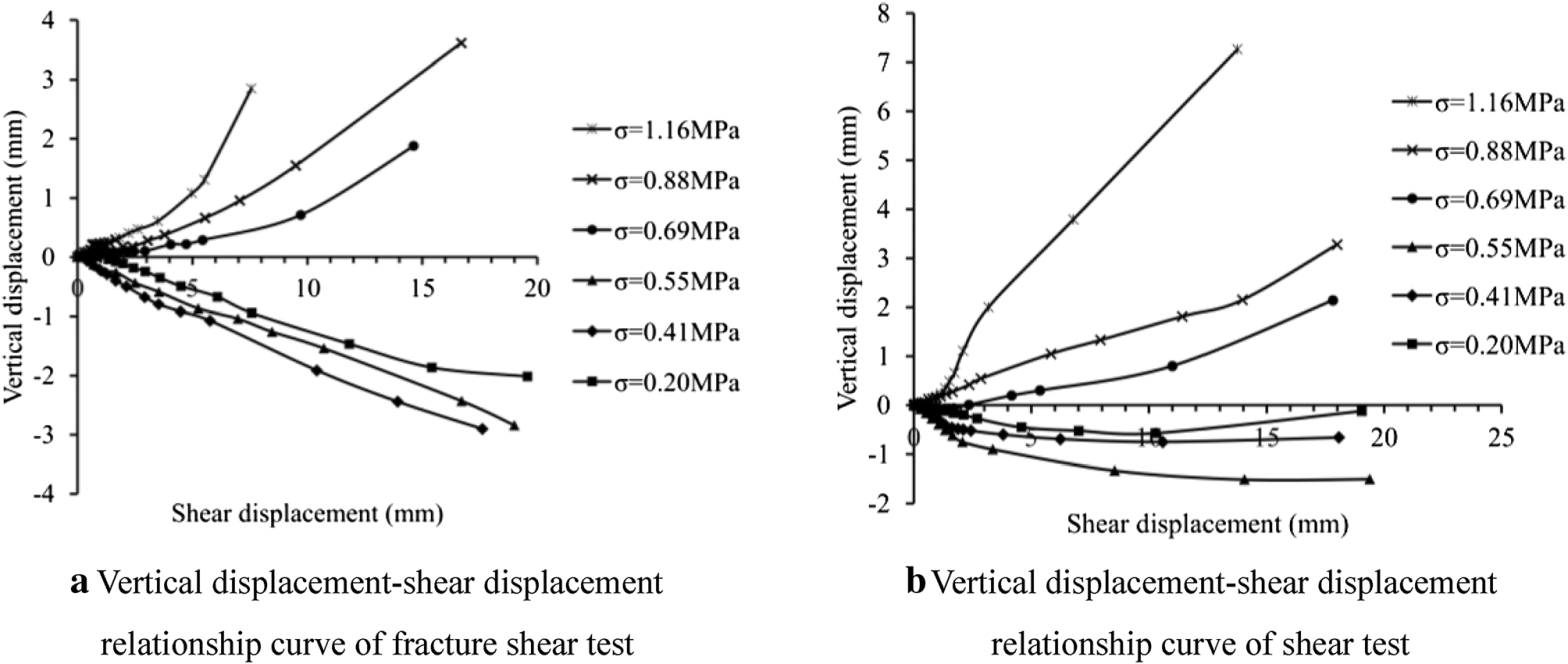
Vertical displacement characteristics of the sample.

During the first shear, samples basically show linear compression deformation vertically as shear displacement increases when normal stress is smaller than 0.55 MPa. The maximum vertical compression deformation rate of Sample 2# is the largest and Sample 1# is the smallest. If normal stress is greater than 0.55 MPa, samples have expansion deformation vertically, which is in non-linear increasing tendency with the shear displacement. As the normal stress increases, expansion deformation degree and rate also increase (Fig. 12a). During the second shear, samples show compression deformation vertically when the normal stress is smaller than 0.55 MPa. The compression deformation decreases with the increase of normal stress. When normal stress is greater than 0.55 MPa, samples show expansion deformation on sheared face, which increases with normal stress (Fig. 12b). Compared with the first shear, the compression deformation is obviously restrained, probably because that sheared face has formed. However, expansion deformation barely changes.

If take the said maximum fluctuation amplitude of sheared face as the width of shear zone, the width increases with positive pressure. In case of low stress, particles in the shear zone breaks under the effect of small scope of shearing. The volume reduction results in the vertical compression deformation. In case of high stress, secondary sheared face (steps generated) enlarges the scope of shearing effect and brings climbing into existence which results in the expansion of shear zone. Shear zone expansion not only happens before sample failure (shear failures of all samples are within the shear displacement of 5 mm), but also gets larger after sample clipping or shearing.

### Ring shear test data

#### Saturated drained shear test

After test begins, pore pressure increases with shear stress. When shear stress is equivalent to shear strength of sample (120 kPa), sample breaks and pore pressure reaches the peak at the moment. Then shear stress drops quickly, pore pressure decreases therewith. After a slight decrease, shear stress goes up again slowly, but pore pressure continuously decreasing until it is down to zero. When no pore pressure exists, shear stress gradually fixes at a constant value, which means that sample has reached its residual shear strength (140 kPa). It could be concluded that sample shear strength might not drop significantly under drainage condition.

**Figure 13:**
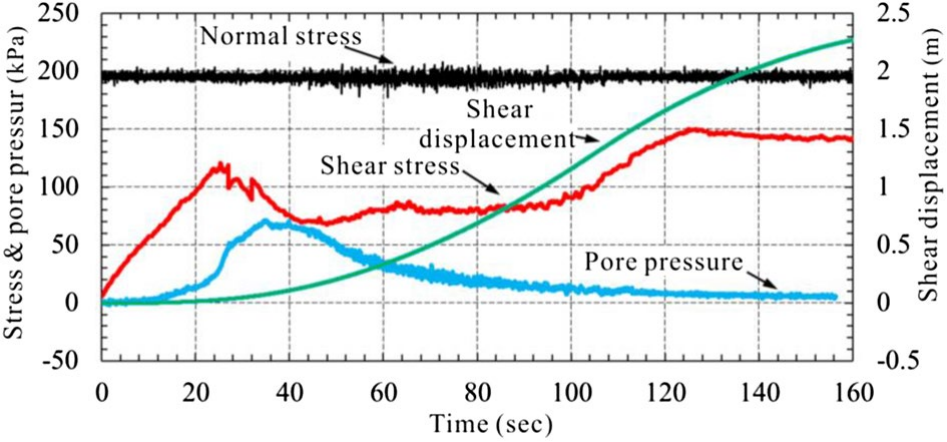
Shear characteristics of saturated drainage.

According to Fig. 13, pore pressure would rise a little bit in the beginning drained shear process, which results in a lower peak shear strength of sample. Shear stress decreases after peak, but gradually restores as pore pressure discharges out. In residual state, shear strength of sample is equivalent to that of dry sample. After sample is broken, growing rate of shear displacement slows down, which means that shear rate slows down as well.

#### Undrained static shear test

Pore pressure of S1 increases slowly with the shear stress. At the moment of 280 s, sample is broken, shear displacement increases, shear stress still increases intermittently with slight increment in pore pressure, which means that shear strength of sample rises with shear speed after failure. After 400 s, shear stress is kept at 205 kPa, with the pore pressure of only 17.2 kPa, pore pressure ratio (pore press/normal stress) of 0.08 and total shear displacement of 4.2 m. Pore pressure of S2 increases quickly with shear stress. Peak strength is reached when shear stress is up to 71.5 kPa. At this moment, sample is broken, shear stress starts to drop quickly, but pore pressure and shear displacement increase quickly. Finally, sample reaches the residual strength with the stable shear stress at 44 kPa, pore pressure at 140 kPa, pore pressure ratio at 0.7 and shear displacement at 2.3 m. Shear stress of S3 increases quickly in linear form. While shear displacement and pore pressure increments are not obvious. Pore pressure even shows negative growth at the moment of 100 s. At 203 s, pore pressure reaches the minimum level at 16.8 kPa and sample shear stress reaches the maximum at 139.6 kPa. Then shear stress drops quickly, pore pressure and displacement rise quickly. At 260 s, shear stress is almost constant at 7.8 kPa, pore pressure at 193 kPa and pore pressure ratio reaches 0.97. Sample is completely liquefied and reaches residual state with the final shear displacement of 7.2 m.

**Figure 14:**
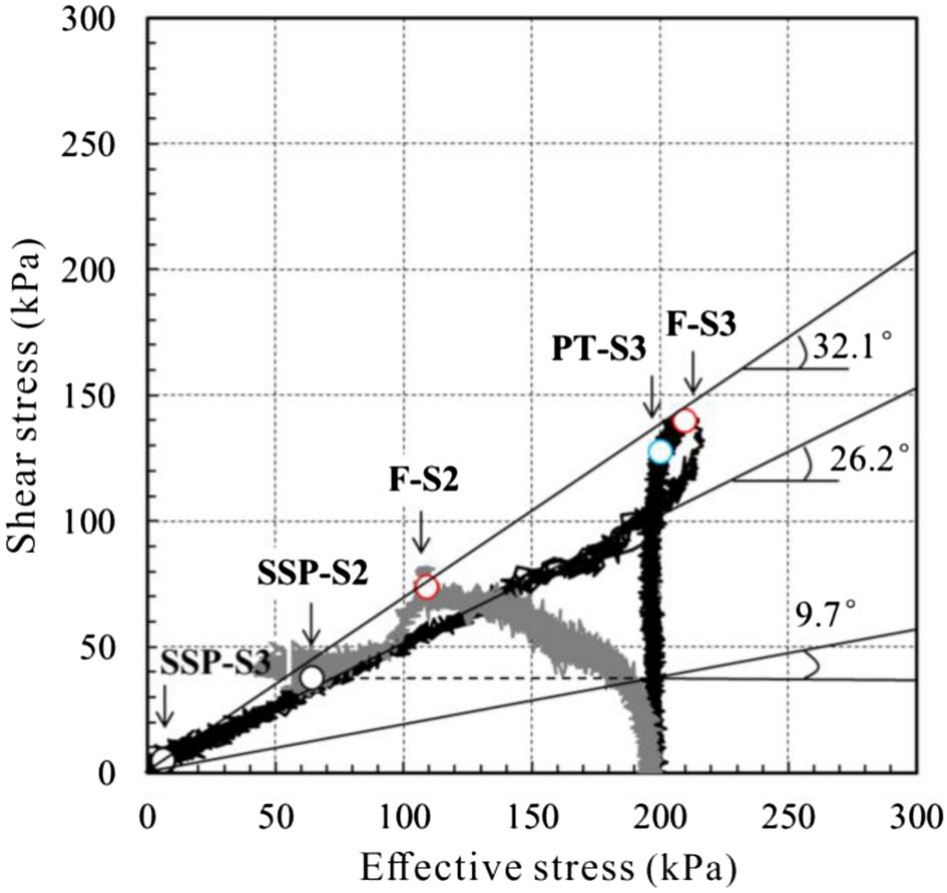
Effective stress paths of S2 and S3.

From the stress route curve (Fig. 14), it can be known that effective stress of S2 drops quickly as shear stress increases, curve moves leftwards. Shear stress starts to go down after reaching the peak (F-S2) and effective stress goes on dropping. The curve finally stops at the steady state point (SSP-S2). For S3, shear stress is in linear increasing at the beginning, while its effective stress nearly unchanges. After passing the state change point (PT-S3), effective stress increases with shear stress, curve starts to move rightwards. When reaching the peak (F-S3), effective stress decreases fast with shear stress. The curve extends leftwards in linear form and finally stops at steady-state point (SSP-S3).

**Figure 15:**
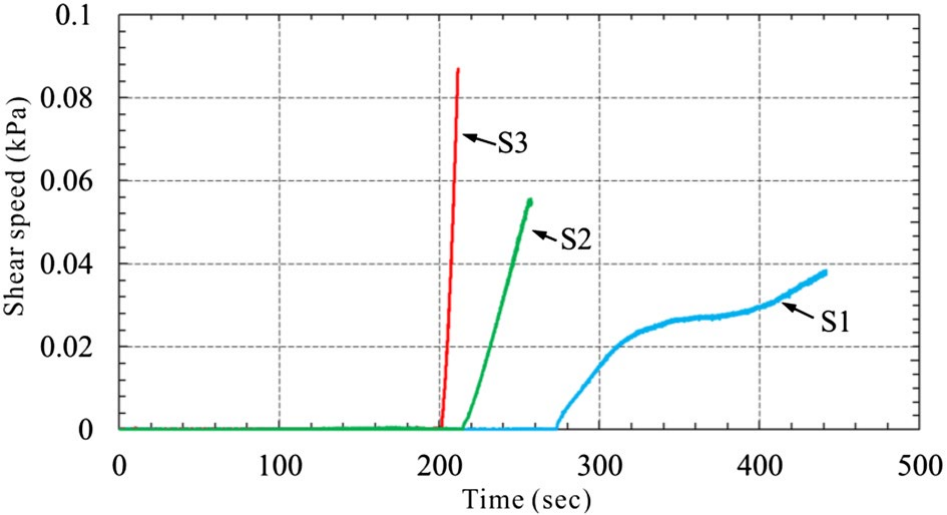
Shear velocity–time curve.

According to the comparison results, S1 cannot be liquefied under undrained conditions and there is no obvious peak strength. Instead of decreasing, shear strength increases with the shear rate. The shear strength of S3 is greater than S2. After peak, pore pressure growing ability of S3 is greater than S2, which means that S3 is much easier to be liquefied than S2, and would get larger shear velocity and acceleration after liquification (Fig. 15).

## Discussion

Taking advantage of the 3 field and indoor testing methods including test at different moisture contents, saturated drainage and undrained tests, clipping and shearing tests, it is discovered that the cohesion scope of fault material is 20–320 kPa and inner friction angle range is 15°–41°. Because of the restrictions of indoor test conditions, the test results may be affected by some factors which shall be recognized when taking the test data for reference.

Indoor samples are disturbed samples taken from the site, hence the original structure of the fault materials must have been destroyed, such as cracks and pores. And the sampling process will also causes further damage to the material. In addition, to make the density of sample close to the original materials, hammering should be done during the sample preparation of medium scale shear test, which may also worsen the damage degree of particles. The damage to the original structure of materials is bound to affect the water holding capacity of materials and might affect the test results of material shear characteristics. For the limited functions of laboratory apparatus, some large particles like particles larger than 20 mm for medium scale test apparatus and ones larger than 2 mm for simple direct shear test apparatus shall be screened out while loading samples to make a difference between samples and original materials. In fact, the fault stress could reach 11 MPa at maximum, but the original stress status of materials still cannot be returned by the test results for the limited range of normal stress applied by the test apparatus^25^. We speculate the shear characteristics of materials under the stress greater than the test stress by fitting. Actually, sampling removes the constraints of original stress on materials. For example, materials breaking and cementation by hydrothermal solution happen in active geological structure movement, while material unloading happen in inactive geological structure movement which gives residual stress to cemented rock mass.

Although more reliable results have been obtained than indoor middle scale shear test and simple direct shear test, in-situ shear test still has limitations. The sample preparation process of shear test removed the stress constraints of rock mass around, which changed the original stress status of materials to some extent. Furthermore, it is hard for the in-situ shear test loading system to give the equivalent normal stress to the DGB landslide. It is impossible to make the test sample completely in conformity with the standard shape (absolute flat on top face, and profile is absolutely vertical to the top face) even though the fault rock is broken, as the broken rock is harder than soil and easy to break up. So test error is inevitable. What’s more important is the environmental influence of groundwater. According to the tunnel excavated on site^10^, the fault material has the characteristics of groundwater storage and migration, and the material is basically in a saturated state. However, the sample preparation process of in-situ shear test has changed the groundwater context, including changing the water content in materials and completely cutting off the groundwater supply. Besides, the deep-buried fault materials in saturated and recharge state should be in an undrained (at least semi-drained) conditions during rapid shear process. But in-situ shear test, groundwater conditions cannot be taken into consideration. Although the original stress environment is difficult to restore and sample error is inevitable, the influence of groundwater to materials could be eliminated by new test means.

**Figure 16:**
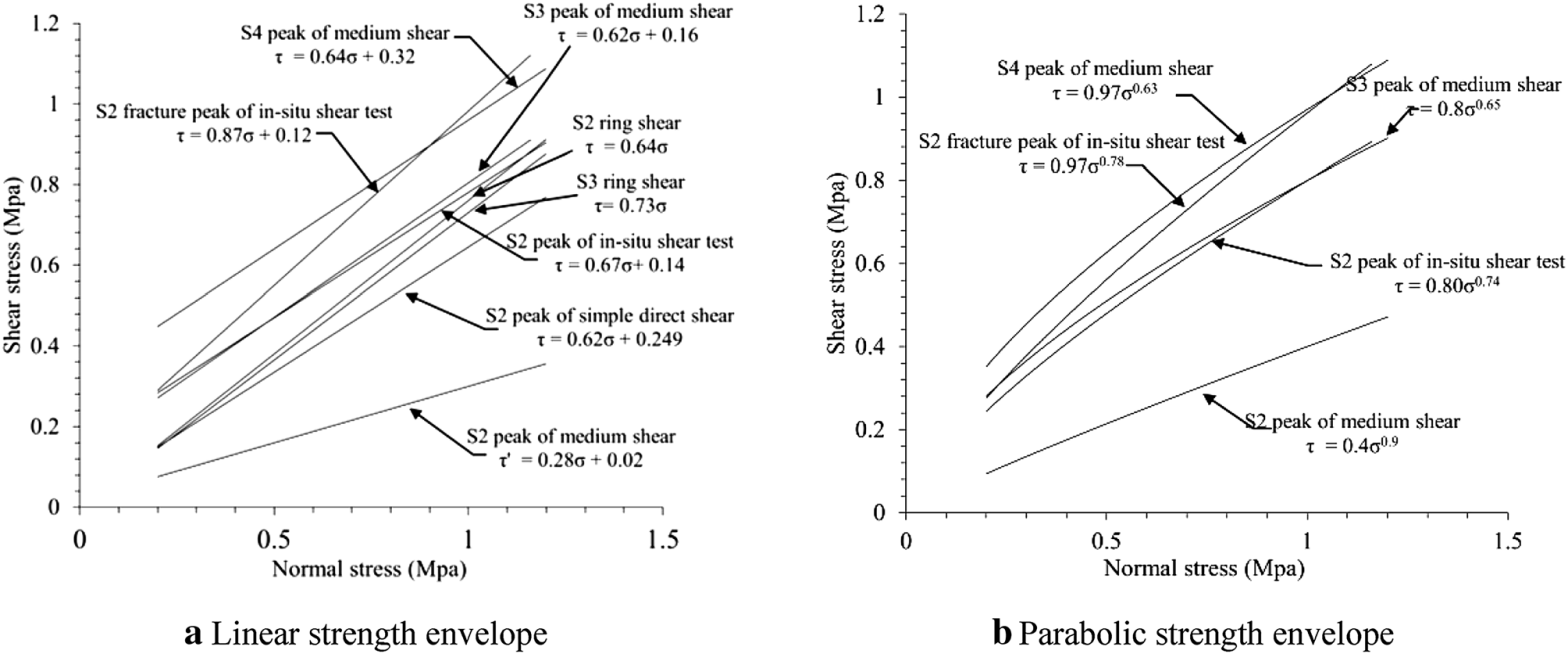
Comparison of shear properties of materials with different methods.

The in-situ shear test peak envelope line of S2 is almost same as that of S3. Compared with the middle scale shear peak envelope lines of S3 and S4, as well as direct shear peak envelope line of S2, the inner friction angles of S2 large shear is almost same as the three, but the cohesion of S2 large shear peak is close to that of S3 middle shear peak, smaller than S4 middle shear and greater than S2 direct shear. Based on all the test results, S2 middle shear shows the smallest inner friction angle (15°) and S2 large shear shows the largest (41°). This might be caused by size effect^26^. The inner friction angle parameters of fault materials has little to do with the results obtained by different test means (32°–36°). In-situ shear test has not only averted the disturbance on samples but also avoided changing the original particle size and grading of materials, so the test results are more reliable (Fig. 16).

**Figure 17:**
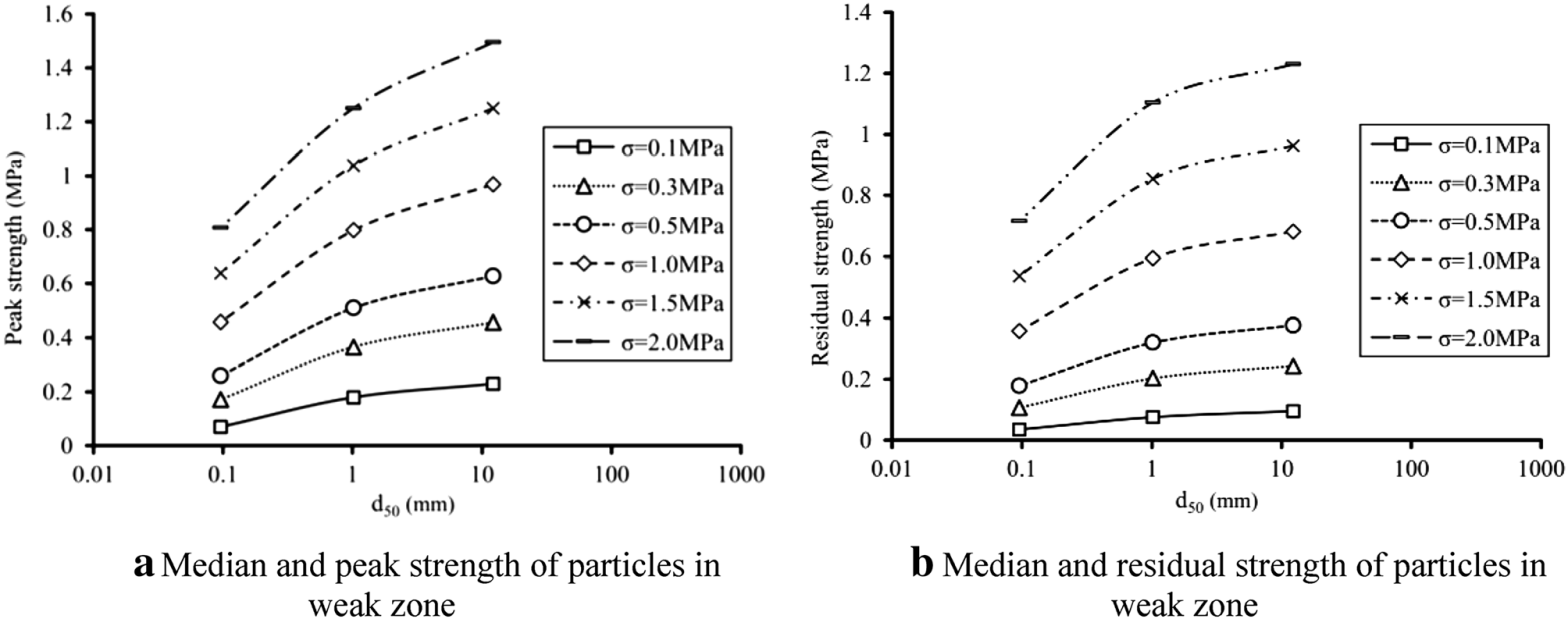
Influence of material crushing degree on shear strength.

Granulometric composition has significant effect on the interbedded strength of fault materials. In this paper, median of the particle distribution curve (d_50_) is taken to represent the crushing degree of particle samples (Fig. 17). The medians of S2, S3 and S4 are 0.09 mm, 1.02 mm and 12.23 mm respectively, which means that d_s2_ < d_s3_ < d_s4_. In addition, peak strength and residual strength of samples under the normal stress of 0.1–2 MPa are worked out by the foregoing strength envelope line formula. Under the same normal stress, both peak strength and residual shear strength increase with the median, which means that different structural zones of fault materials are different in shear strength. The more seriously damaged structural zone or structural rock, the lower the shear strength. Besides, the influence of particle size on strength grows with the normal stress. Taking everything into consideration, it can be inferred that shear strength of material in mylonitic zone is smaller than breccia zone under the original stress of DGB landslide without taking water influence into consideration, namely $\tau_{s2} < \tau_{s3} < \tau_{s4}$.

What needs to be noted that DGB landslide would not lose stability all of a sudden in dry conditions as water is an important factor to reduce the Coulomb friction of materials. All the tests are conducted in dry environment. All inner friction angles are larger than the apparent dip (16°) of DGB landslide in sliding direction, which means that material shear failure would never take place in this case. However, as previously mentioned, inner friction angle has reduced to 11° when water content of S1 reaches 16%. In saturated undrained conditions, the inner friction angle of S2 could drop to 9.7° and S3 could down to 0, and their inner friction angles could decrease to 23.1° and 4.2° in saturated undrained dynamic shear conditions. The analysis results show that materials of both mylonitic zone and breccia zone are of relatively high liquefaction ability. Under the action of earthquake, the fault material is subjected to strong tension, compression and shear force. Due to the influence of groundwater, the pore pressure of the sliding zone mylonitic material and breccia material with high liquefaction ability increases, liquefies, and the internal friction angle is significantly reduced, and the shear strength decreases rapidly, which makes the landslide start suddenly.

**Figure 18:**
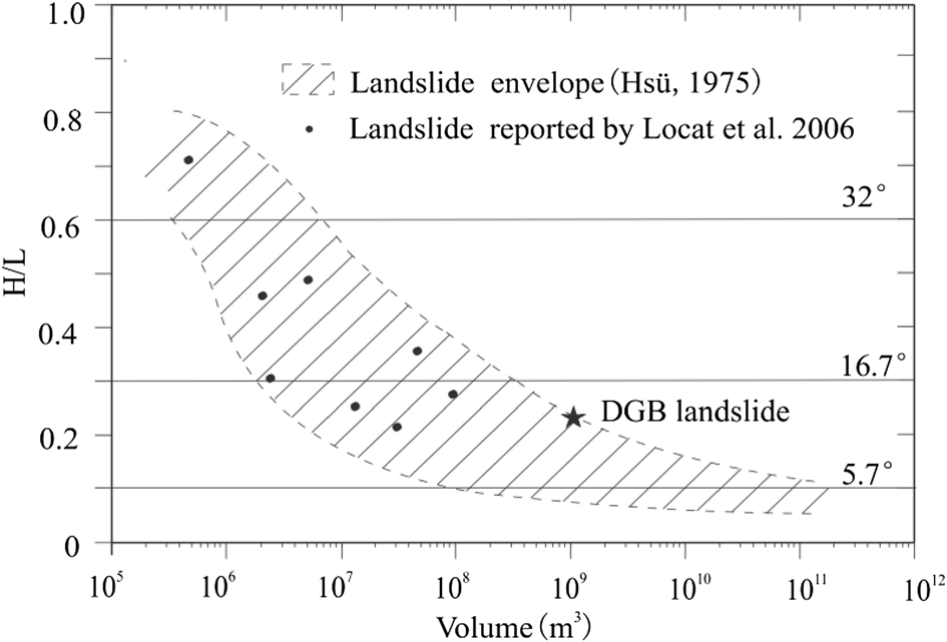
Plot of H/L versus volume for rock avalanches. The lower and upper broken lines represent landslides that show maximum and minimum mobility, respectively, with regards to their volume (modified after Hsü^28^).

After doing tri-axial compression test on materials in cataclinal fault, Zhu et al. declared that the materials were of relatively high liquefaction potential^13^. Cui et al. mainly focused on studying the influence of geological structure on DGB landslide^1^. Test data in this paper have supported their opinions on super pore water pressure from shear behavior point of view. The bedding fault of (at least) 1.8 km possesses very stable attitude, having binding force on DGB landslide motion in early period. Shear softening enables bedding fault to capture the majority of shear strain in motion, so as to change the structure of rock above fault into approximately rigid structure and reduce the breaking degree. Equivalent friction coefficient (f) is widely used to estimate landslide fluidity by defining the rate between the height (H) of the highest point on separation edge and the horizontal projection distance (L) from the mention point to landslide deposit end^27^. Material displacement changing with its friction behavior is characterized by motion angle (tan^–1^(H/L)). Wherein H is about 32° or larger (H/L ≥ 0.6). If performance of similar fluid is reached, H/L would be low to 0.1 (about 6°). For DGB landslide with the L of 3.8 km and f of 0.23 (tan (13°)) showing very large moving ability among global large scale landslides (Fig. 18), it is believed that low friction of the bedding fault is the main reason.

## Data Availability

All data will be available on reasonable request from corresponding author.
